# An Evolutionary Model of Bounded Rationality and Intelligence

**DOI:** 10.1371/journal.pone.0050310

**Published:** 2012-11-21

**Authors:** Thomas J. Brennan, Andrew W. Lo

**Affiliations:** 1 Northwestern University School of Law, Chicago, Illinois, United States of America; 2 MIT Sloan School of Management, CSAIL, and EECS, Cambridge, Massachusetts, United States of America; 3 AlphaSimplex Group, LLC, Cambridge, Massachusetts, United States of America; Tel Aviv University, Israel

## Abstract

**Background:**

Most economic theories are based on the premise that individuals maximize their own self-interest and correctly incorporate the structure of their environment into all decisions, thanks to human intelligence. The influence of this paradigm goes far beyond academia–it underlies current macroeconomic and monetary policies, and is also an integral part of existing financial regulations. However, there is mounting empirical and experimental evidence, including the recent financial crisis, suggesting that humans do not always behave rationally, but often make seemingly random and suboptimal decisions.

**Methods and Findings:**

Here we propose to reconcile these contradictory perspectives by developing a simple binary-choice model that takes evolutionary consequences of decisions into account as well as the role of intelligence, which we define as any ability of an individual to increase its genetic success. If no intelligence is present, our model produces results consistent with prior literature and shows that risks that are independent across individuals in a generation generally lead to risk-neutral behaviors, but that risks that are correlated across a generation can lead to behaviors such as risk aversion, loss aversion, probability matching, and randomization. When intelligence is present the nature of risk also matters, and we show that even when risks are independent, either risk-neutral behavior or probability matching will occur depending upon the cost of intelligence in terms of reproductive success. In the case of correlated risks, we derive an implicit formula that shows how intelligence can emerge via selection, why it may be bounded, and how such bounds typically imply the coexistence of multiple levels and types of intelligence as a reflection of varying environmental conditions.

**Conclusions:**

Rational economic behavior in which individuals maximize their own self interest is only one of many possible types of behavior that arise from natural selection. The key to understanding which types of behavior are more likely to survive is how behavior affects reproductive success in a given population’s environment. From this perspective, intelligence is naturally defined as behavior that increases the probability of reproductive success, and bounds on rationality are determined by physiological and environmental constraints.

## Introduction

Most economic theories assume that individuals behave rationally, maximizing their own self-interest subject to resources constraints. This framework has led to numerous breakthroughs in economic science, including expected utility theory [1] (an axiomatic formulation of rational behavior under uncertainty), the notion of “rational expectations” [2] (individual expectations are formed to be mutually consistent with those arising from economic equilibria), and the “efficient markets hypothesis” [3], [4] (market prices fully reflect all available information). While other alternatives have been proposed, such as heuristic approximation (“satisficing”) and bounded rationality [5], the vast majority of current economic models still assume the ideal of a fully rational and optimizing individual, often referred to as *Homo economicus*. The influence of this paradigm goes far beyond academia–it underlies current macroeconomic and monetary policies, and has also become an integral part of the rules and regulations that govern financial markets today [6], [7].

However, there is mounting empirical and experimental evidence, including the recent financial crisis, suggesting that humans do not always behave rationally, but often make seemingly random and suboptimal decisions [8]. These behavioral anomalies are especially pronounced when elements of risk and probability are involved, and two of the most well-known are probability matching [9], [10] (the tendency to choose randomly between heads and tails when asked to guess the outcomes of a series of independent biased-coin tosses, where the randomization matches the probability of the biased coin), and loss aversion [11] (the tendency to take greater risk when choosing between two potential losses, and less risk when choosing between two potential gains). Both behaviors are clearly suboptimal from the individual’s perspective, yet they have been observed in thousands of geographically diverse human subjects over several decades. Such anomalous behaviors have also been observed in many non-human subjects including ants [12]–[15], bees [16]–[18], fish [19], [20], pigeons [21], [22], and primates [23], which suggests that they may have a common and ancient origin, and an evolutionary role that belies their apparent shortcomings.

Accordingly, several evolutionary models have been proposed to explain these counterintuitive behaviors [24]–[26], as well as a variety of other social customs including altruism, cooperation, subterfuge, self-deception, kin selection, and reciprocity [27]–[31]. The fields of sociobiology and, more recently, evolutionary psychology have expanded the reach of evolution to even broader domains such as language, culture, and religion [29], [32]–[35]. However, it is unclear how these behaviors relate to standard economic theories of individual rationality, why they emerge in some instances and not others, and what part intelligence plays in such behaviors.

The economics literature has also considered evolutionary arguments, primarily through the natural selection of utility functions that individuals maximize [36]–[47]. In financial-market contexts, the evolution of trading strategies [48]–[57] and supply/demand functions [58]–[60] have also been considered. However, the starting point for these models is considerably more sophisticated behavior than what we propose in our framework. In particular, utility maximization, the existence of excess demand functions, or specific trading strategies already assume a certain degree of goal-seeking behavior and intelligence, which are traits we derive in a much simpler, less structured binary choice model [26]. From purely mindless acts of choosing between two alternatives, we show that natural selection alone is capable of generating very specific behavioral patterns such as risk aversion, loss aversion, and mixed strategies. More importantly, with this primitive framework, we are able to derive the beginnings of what can plausibly be construed as intelligent behavior and how such intelligence is naturally bounded by environmental and physiological constraints.

The key feature is the interaction between individual behavior and the stochastic environment in which reproductive success is determined, and the difference between idiosyncratic and systematic risk is of central importance as documented in many earlier studies [24], [26], [38], [40], [46]. If all individuals behave identically and deterministically, choosing the course of action that leads to the highest expected number of offspring, this can lead to extinction if reproductive uncertainty is perfectly correlated across individuals in a given generation, i.e., if all individuals occupy the same ecological niche. For example, if all individuals choose to forage in the same higher-yielding patch, the first time that patch becomes barren, the entire population will be wiped out. In such environments, randomizing behavior such as Herrnstein’s Law [10] may be favored by natural selection over any type of deterministic behavior. What we observe as irrational behavior may indeed be irrational from the individual’s perspective, but not from the population’s perspective and it is the latter that is the outcome of natural selection. However, if reproductive success is statistically independent across individuals in a given generation–corresponding to situations in which each individual occupies its own unique niche–we show that natural selection favors individually optimal deterministic behavior instead.

Such a framework provides a natural definition of “intelligence”: any behavior that is positively correlated with reproductive success. If achieving such correlation imposes biological costs on an individual–for example, because it requires attention, memory, planning, and other cognitive faculties–these costs imply an upper bound on the degree of intelligence that emerges through selection. This yields an evolutionary foundation for “bounded rationality” [5]–a heuristics-based model of behavior–as well as a reconciliation between rational economic models and their behavioral violations. Seemingly irrational behavior may be irrational from the individual’s perspective but not necessarily from the population perspective.

## Model

Consider a population in which each individual (not necessarily human) in a given generation 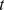 is faced with a single decision in its lifetime, to choose action 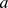 or 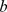, and this choice implies a certain number of offspring 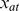 or 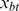, respectively, where 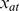 and 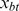 are random variables with joint distribution function 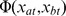. Let individual 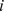
’s behavior be represented by a binary variable 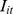 which equals 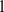 if 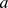 is chosen and 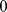 if 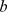 is chosen. Suppose that 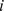 chooses 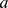 with probability 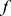 and chooses 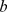 with probability 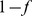 where the probability 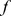 is any value between 0 and 1, including the two endpoints (thus capturing purely deterministic behavior as well). Then 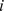
’s behavior is given by the following Bernoulli random variable 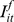:
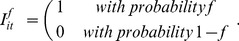
(1)The parameter 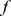 represents the behavioral “phenotype” of an individual, and we assume that this behavior is completely “mindless” in the sense that the individual’s decision 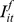 is statistically independent of any other variables in its environment, including the behaviors of others and the outcomes 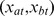. The assumption of independence also implies the absence of any strategic interactions between individuals, since 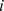
’s choice has no impact on 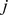
’s reproductive outcomes.

If we assume that the offspring of type-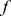 individuals are also of the same type, and we start with an equal number of all types of individuals in the population, we can explore the evolution of behavior by identifying the value of 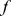 that exhibits the highest geometric growth rate (or “fitness”), which we denote by 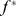. Over time, 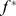 individuals will dominate the population at an exponentially fast rate, hence the behavior 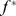 will have “emerged” through the forces of natural selection. We call 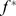 the “growth-optimal” behavior to emphasize this fact.

The particular value of 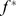 depends critically on the properties of 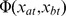, which is a highly compact representation of the biological features of the individual, its random environmental circumstances, and the uncertain impact of behavior on fecundity. Although such a model of evolution, in which individuals live for one period and engage in asexual reproduction with no mutation, is clearly stylized, it does capture the essence of how natural selection shapes behavior. Extensions to biologically more realistic features such as imperfect hereditary transmission of 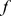, sexual reproduction, and multiple rounds of offspring within a single lifetime can easily be accommodated via constraints on 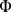 and more sophisticated relationships between the 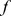 of an offspring and its parents, but at the expense of analytical tractability and transparency.

### Evolutionary Origin of Behavior

Despite the simplicity of this framework, its behavioral implications are surprisingly rich. Suppose we assume that:

(A) 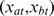 is independently and identically distributed (IID) from one generation to the next, *identically distributed* across individuals 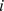 within a given generation 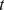, and independent of all other random variables including 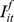 for all 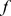 and 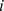.

This assumption allows us to derive a simple expression for the population of type-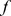 individuals in any given generation. If 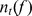 is the number of individuals of type 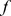 in the population in generation 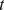, then we have the following recursive expression that captures population growth:
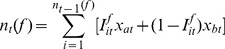
(2)where 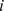 indexes all individuals of type 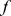 in the previous generation 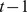.

The assumption that 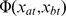 is identically distributed across all individuals within a given generation implies that these individuals are part of the same ecological niche and will produce the same number of random offspring 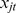 if they choose action 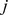, 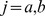. This assumption is implicitly reflected in the fact that 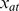 and 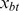 do not require subscript 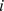
’s because they are identical across all individuals 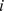 in any generation 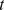. Therefore, (2) may be written as:
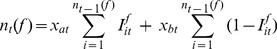
(3)and the Law of Large Numbers implies that the geometric growth rate of each subpopulation of type 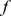 converges in probability to the following limit (see Text S1):
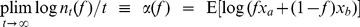
(4)where “
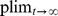
” denotes convergence in probability and we have omitted the 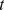 subscript without loss of generality because 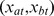 are IID across generations.

By maximizing the growth rate 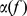 with respect to 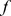, we can determine the behavior 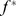 that emerges through natural selection. The maximum is given by:
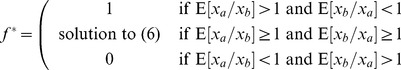
(5)where 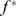 is defined implicitly in the second case of (5) by:
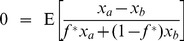
(6)and the expectations in (5) and (6) are with respect to the joint distribution 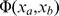.

The solution has three parts. We find that 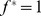 if 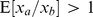 and 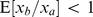, where these inequalities imply that the reproductive yield of 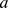 is unambiguously higher than that of 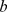. Conversely, 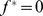 if both inequalities are reversed, in which case the reproductive yield of 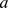 is unambiguously lower than that of 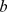. However, if 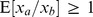 and 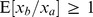, then 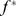 is strictly greater than 0 and less than 1, and is given by the value that satisfies the equality (6). In this case, because the reproductive yield of 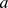 neither dominates nor is dominated by that of 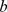, the behavior that yields the fastest growth rate involves randomizing between the two choices with probability 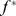, where 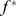 is the value that equates the expected ratio of the number of offspring from each choice to the average number of offspring across the two choices.

This result is surprising to economists because it seems inconsistent with the maximization of self-interest, as well as the deterministic behavior implied by expected utility theory [1]. Suppose 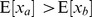 and 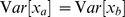 so that action 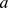 leads to a larger number of offspring on average for the same level of risk; from an individual’s perspective, the “rational” action would be to always select 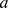, 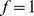. However, such individually rational behavior will eventually be dominated by the faster-growing 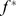-types, hence it cannot persist over time. The growth-optimal behavior 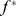 may be viewed as a primitive version of “altruism”, i.e., behavior that is suboptimal for the individual but which promotes the survival of the population.

### A Simulation Experiment

The emergence of behavior is most easily seen through a simple simulation of the binary-choice model in a specific context where probability-matching behavior arises. Consider an environment in which it is sunny and rainy with probability 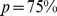 and 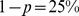, respectively. Individuals must decide where to build their nests, in the valley (choice 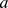) or on a plateau (choice 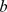). During sunny days, nesting on a plateau will yield 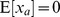 offspring because of the heat of the sun and lack of water, whereas nesting in the valley yields 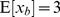 offspring because of the valley’s shade and the streams that run through it. During rainy days, the exact opposite outcomes are realized: nesting in the valley yields 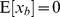 because the valley will flood, drowning all offspring, but nesting on a plateau yields 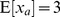 because the rain clouds provide both water and protection from the sun. In this environment, the behavior that maximizes the survival probability of an individual’s offspring is to choose 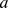 all the time (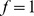) since the probability of sunshine is 75%. However, such behavior cannot survive–the first time it rains, all individuals of type 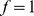 will be eliminated from the population. In fact, the behavior yielding the highest growth rate is 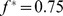; hence, “probability matching” behavior, also known as “Herrnstein’s Law”, [10], [24], [26] is evolutionarily dominant in this special case.

For other values of the outcomes of 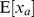 and 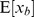, 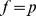 may not yield the highest rate of growth, but 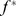 can nevertheless be strictly greater than 0 and less than 1, so that randomizing behavior will still persist. When faced with environmental randomness that affects the entire population in the same manner (recall our “single-niche” assumption), and where the type of randomness yields extreme outcomes for different behaviors, deterministic behavior cannot survive because at some point, an extreme outcome will occur, wiping out that subpopulation. The only way to survive is to randomize, and the subpopulation that grows fastest in this type of environment is one in which 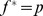. For concreteness, Table 1 contains a numerical simulation of this example.

This simple example can be easily generalized to any arbitrary number of offspring for both choices [26]:
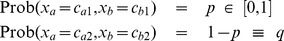
(7)where we assume that 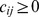 and 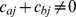, 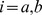 and 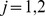. The condition 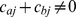 rules out the case where both 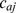 and 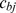 are 0, in which case the binary choice problem becomes degenerate because both actions lead to extinction hence the only choice that has any impact on fecundity is in the non-extinction state, and the only behavior that is sustainable is to select the action with the higher number of offspring.

**Table 1:** Simulated population sizes for binary-choice model with five subpopulations in which individuals choose 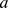 with probability 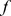 and 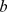 with probability 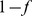, where 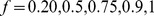, and the initial population is 10 for each 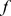.

Generation	f = .20	f = .50	f* = .75	f = .90	f = 1
1	21	6	12	24	30
2	12	6	6	57	90
3	6	12	12	144	270
4	18	9	24	387	810
5	45	18	48	1,020	2,430
6	96	21	108	2,766	7,290
7	60	42	240	834	21,870
8	45	54	528	2,292	65,610
9	18	87	1,233	690	196,830
10	9	138	2,712	204	590,490
11	12	204	6,123	555	1,771,470
12	36	294	13,824	159	5,314,410
13	87	462	31,149	435	15,943,230
14	42	768	69,954	1,155	0
15	27	1,161	157,122	3,114	0
16	15	1,668	353,712	8,448	0
17	3	2,451	795,171	22,860	0
18	3	3,648	1,787,613	61,734	0
19	9	5,469	4,020,045	166,878	0
20	21	8,022	9,047,583	450,672	0
21	6	12,213	6,786,657	1,215,723	0
22	0	18,306	15,272,328	366,051	0
23	0	27,429	34,366,023	987,813	0
24	0	41,019	77,323,623	2,667,984	0
25	0	61,131	173,996,290	7,203,495	0

Reproductive uncertainty is systematic and also binary, with 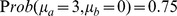 and 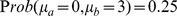. In this setting, probability matching 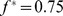 is the growth-optimal behavior.

The growth-optimal behavior in this case will depend on the relation between the probability 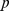 and the relative-fecundity variables 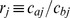 for each of the two possible states of the world 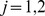. Applying (5) under the distribution (7) for 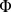 yields the following growth-optimal behavior 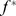:
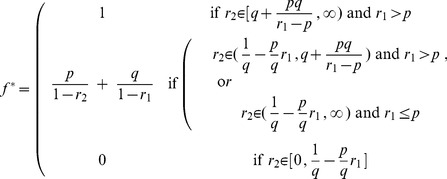
(8)Since 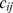 may be 0, the ratios 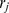 may be infinite if a finite numerator is divided by 0, which poses no issues for any of the results in this paper as long as the usual conventions involving infinity are followed. The ambiguous case of 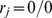 is ruled out by the condition 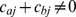.

**Figure 1:**
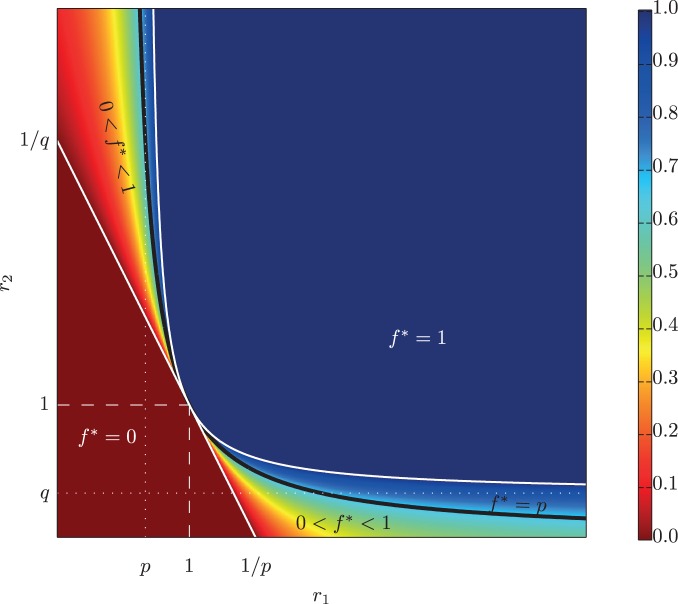
Regions of the 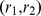-plane that imply deterministic (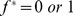) or randomizing (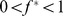) behavior, where 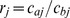 measures the relative fecundities of action 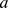 to action 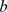 in the two states 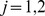. The asymptotes of the curved boundary line occur at 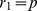 and 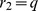. Values of 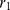 and 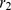 for which exact probability matching is optimal is given by the solid black curve.

Figure 1 illustrates the values of 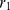 and 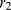 that yield each of the three types of behaviors in (8). When 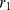 and 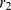 satisfy the condition:
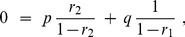
(9)exact probability matching behavior arises, and the solid black curve in Figure 1 illustrates the locus of values for which this condition holds. The horizontal asymptote of the curve occurs at 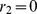, so as 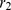 tends toward zero and 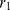 becomes relatively large, exact probability matching will be optimal (note that the asymmetry between 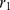 and 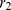 is due entirely to our requirement that 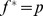 and 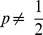). However, values of 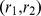 off this curve but still within the shaded region imply random behavior that is approximately–but not exactly–probability matching [26], providing a potential explanation for more complex but non-deterministic foraging patterns observed in various species [12]–[14], [17], [18].

### Idiosyncratic Reproductive Risk

Now suppose we change our assumption that individuals all belong to the same ecological niche, and assume instead that:

(B) 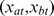 is IID across individuals in each generation, as well as from one generation to the next, and independent of all other random variables including 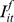 for all 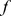 and 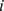.

This corresponds to the situation in which each individual occupies its own unique niche, receiving a separate and independent random draw 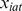 or 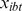 from the same respective distributions as others. In this case, the Law of Large Numbers applies across individuals within each generation as well as over time, and the growth rate of type-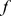 individuals is given by:
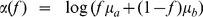
(10)where 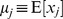, 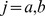. This function contains no random variables and attains its maximum at 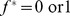, depending on whether 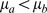 or 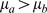, respectively. Because individuals within any given generation are already well diversified across statistically independent niches, they can all engage in identical behavior–individually optimal behavior–without the risk of extinction.

When Nature yields systematic environmental shocks to an entire population’s reproductive success, the population must engage in random behavior to ensure that some of its members will survive. However, when Nature imposes idiosyncratic shocks across the population, deterministic behavior can persist because the chances of all individuals experiencing bad draws becomes infinitesimally small as the population size grows. This distinction between systematic and idiosyncratic environments is the key to reconciling seemingly irrational behavior with *Homo economicus*: the former emerges from systematic environments, and the latter from idiosyncratic ones. Apparently, “Nature abhors an undiversified bet”, hence the type of environmental risk to fecundity determines the type of behavior that has greatest fitness. This observation has profound consequences for behavior, including a natural definition of intelligent behavior and bounded rationality.

## Results

Using the binary-choice framework, natural definitions of intelligence and bounded rationality follow directly. Recall that the individuals in our model are mindless in the sense that their behaviors are assumed to be statistically independent of all other variables. Suppose we relax this assumption by allowing individual decisions to be correlated with other variables such as 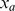 and 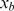:

(C) Let 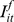 be correlated with 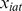 and 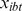, and define 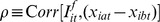 which is assumed to be fixed over generations 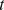.

Correlation between actions and reproductive success is the essence of what we mean by “intelligent behavior.”

### Intelligence: An Evolutionary Definition

As before, consider an initial population with equal numbers of individuals of all types 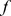, and with arbitrary correlations between 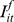 and 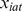 and 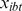 so that no single value is over-represented. Applying the Law of Large Numbers, we see that the growth rate for individuals of type 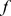 with correlations.
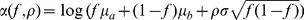
(11)where 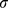 is the standard deviation of 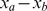. In this case, the growth rate is equal to the growth rate of the mindless population plus an extra term 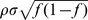 that reflects the impact of correlation between an individual’s decision and the number of offspring. Several implications follow immediately from this expression.

First, subpopulations with negative correlation between behavior and 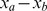 clearly cannot survive in the long run; their growth rates are less than the no-correlation case, and correspond to counter-productive behavior in which decisions coincide with lower-than-average reproductive outcomes more often than not, i.e., choosing 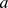 when 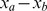 is lower than average and choosing 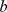 when the reverse is true. By the same logic, subpopulations with positive correlation will grow faster, and individuals with the highest correlations 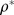 will dominate the population. We suggest that these cases may be considered primitive forms of “intelligence”–behavior that yields improved fitness.

The subpopulation with the largest 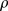 will grow fastest and come to dominate the population. For example, certain senses such as hearing and eyesight are so highly correlated with reproductive success that they become universally represented in the population. By optimizing 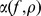 with respect to 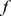 and 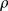 to yield 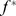 and 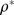, we arrive at the growth-optimal level of intelligence and behavior that emerges from the population (see Text S1):
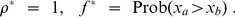
(12)Perfect positive correlation always dominates imperfect correlation, and despite the presence of idiosyncratic reproductive risk, the growth-optimal behavior involves probability matching, albeit a different kind in which 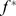 matches the probability of 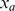 exceeding 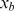.

### Bounded Rationality

If there is no biological cost to attaining 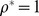, then perfect correlation will quickly take over the entire population, and because we have assumed no mutation from one generation to the next, all individuals will eventually possess this trait. However, it seems plausible that positive correlation would be associated with positive cost. For example, by using certain defense mechanisms such as chemical repellants or physical force, animals can fend off predators. This behavior increases their expected number of offspring, but the physiological cost of defense may decrease this expectation, hence the evolutionary success of such behavior depends on the net impact to fitness. If we define a cost function 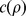, then we can express the “net” impact of correlation by deducting this cost from the correlation itself to yield the following asymptotic growth rate of type-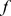 individuals:
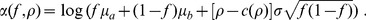
(13)With plausible conditions on 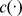 and 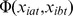, there is a unique solution 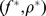 to 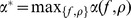. Because 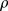 is subject to a nonlinear constraint that depends on 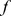, explicit expressions for 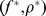 are not as simple as the no-intelligence case (see Text S1 for details). However, the structure of the solution is qualitatively identical and intuitive: 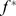 reduces to three possibilities, either 0 or 1 if correlation is too “expensive” to achieve, or the probability-matching solution 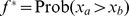 if the cost function 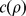 is not too extreme. This growth-optimal solution is an example of bounded rationality–bounded in the sense that higher levels of 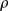 might be achievable but at too high a cost 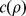. The behavior that eventually dominates the population is good enough, where “good enough” now has a precise meaning: they attain the maximum growth rate 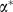. In other words, 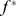 is an example of satisficing.

If the cost of intelligence is influenced by other biological and environmental factors 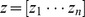, then the multivariate cost function 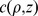 will almost certainly induce a multiplicity of solutions to the growth-optimization problem. This implies a multitude of behaviors and levels of intelligence that can coexist because they yield the same maximum population growth rate 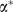. The set of behaviors 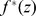 and intelligence 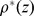 that emerge from the population will be a function of 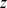 and given implicitly by the solution to 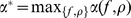. This provides a direct link between adaptive behavior and the environment, which is the basis for models of social evolution and evolutionary psychology [27], [29], [61], [62].

## Discussion

The simplicity and generality of our framework suggest that the behaviors we have derived are likely to be quite primitive on an evolutionary timescale, and that most species will have developed the necessarily biological apparatus to engage in such behavior under the right environmental conditions.

However, evolution can also produce more sophisticated behaviors such as overconfidence [63], altruism and self-deception [61], and state-dependent strategies like the Hawk-Dove game [64], which emerge as a result of more complex environmental conditions. For example, if we assume that one individual’s action can affect the reproductive success of another individual, e.g., 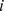
’s fecundity is influenced by 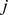
’s selection of 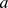 or 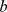, individuals engaging in strategic behavior will reproduce more quickly than those with simpler behaviors such as probability matching or loss aversion. If the actions of individuals in the current generation can affect the reproductive success of individuals in future generations, even more complex dynamics are likely to emerge as in the well-known overlapping generations model [65]. In a resource-constrained environment in which one individual’s choice can affect another individual’s reproductive success, strategic interactions such as reciprocity and cooperation will likely emerge within and across generations [28], [31]. Other extensions of the binary-choice framework include time varying environmental conditions 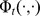, mutation through sexual reproduction, and multiple reproductive cycles within a single lifetime (iteroparity). Each of these extensions captures more realistic aspects of human behavior and taken together, they may provide aggregate measures of systemic risk and financial crisis [66].

In this paper, we have purposefully assumed a much simpler structure, including an unconstrained stable stochastic environment with no strategic considerations, so as to determine what types of behavior are truly primitive. Even in such a simple setting, we find a surprisingly complex and subtle range of behaviors–behaviors that do not always conform to common economic intuition about rationality–can arise and persist via natural selection. Simon [67] illustrated this principle vividly with the example of a single ant traversing a mixed terrain of sand, rocks, and grass. The ant’s path seems highly complex, but the complexity may be due more to the environment than the ant’s navigational algorithm.

This perspective has received more recent support from the discovery of remarkably sophisticated social behavior among bacteria [68–76]. There is little doubt that an individual bacterium is mindless, yet colonies of such bacteria engage in seemingly intelligent behavior such as competition, collaborative foraging, and cell-to-cell chemotactic and physical communication. Such behavior can ultimately be traced to genetic structures [66], but the complementary approach of linking behavior directly to reproductive outcomes may yield additional insights into the common evolutionary origins of certain behaviors.

While it is nearly self-evident that the critical determinant of which behavior emerges from a given population is the interaction between the biological features of the individuals and the nature of the environment, our simple framework shows just how powerful environmental forces can be in shaping fundamental aspects of decisionmaking. If we seek to understand the origin of intelligence and the limits of rational behavior, we may find useful answers by studying current and past environments in addition to studying our genes.

## Supporting Information

Figure S1Values of 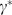 and 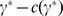 as functions of 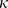, the cost of intelligence parameter in equation (26) of Text S1.(EPS)

Figure S2Values of 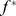 for particular values of 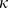 and 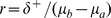. The region toward the upper left corresponds to relatively costly intelligence and deterministic behavior of the form 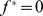. The region toward the lower right corresponds to relatively cheap intelligence and probability matching of the form 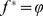. On the line between the two large regions, any value for 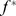 between 0 and 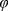 is optimal.(EPS)

Text S1 Proofs and derivations of all the results in the main text are provided in this document.
(PDF)
