# Supplementary material for: An Evolutionary Model of Bounded Rationality and Intelligence
Source: PLoS One. 2012 Nov 21;7(11):e50310. doi: 10.1371/journal.pone.0050310 (PMC3504016; doi:10.1371/journal.pone.0050310)
Supplement: Text S1 — Proofs and derivations of all the results in the main text are provided in this document. (PDF) [file pone.0050310.s003.pdf]

An Evolutionary Model of Bounded Rationality and Intelligence: Supporting Text S1

Thomas J. Brennan[1] and Andrew W. Lo[2,3]

[1]*Northwestern University School of Law, 375 East Chicago Avenue Chicago, IL 60611–3069, USA*

[2]*MIT Sloan School of Management, CSAIL, and EECS 100 Main Street, E62–618, Cambridge, MA 02142–1347, USA*

[3]*AlphaSimplex Group, LLC, 1 Cambridge Center Cambridge, MA 02142–1347, USA*

1 The General Model

We first present our general model, which encompasses the possibility of variation in outcomes across individuals within a generation, as well as across generations, and also the possibility of intelligent behavior. In the following sections we consider several special cases giving rise to the results stated in the accompanying paper.

Each individual in a population is faced with a single decision in its lifetime, choosing action a or b, and this choice results in a certain number of offspring, x_a or x_b, respectively. The quantities x_a and x_b are random variables with a joint distribution $\Phi(x_a, x_b)$. The behavior of individual i is represented by a $0/1$ Bernoulli trial, I, with probability f, i.e., a is chosen with probability f (in which case $I = 1$), and b is chosen with probability $1 - f$ (in which case $I = 0$). When we wish to specify the outcomes applicable to a particular individual, i, for any of these variables, we add a subscript i. Similarly, when we wish to specify a particular generation, t, we add the subscript t.

We assume that an individual with a choice function I has offspring with the identical choice function I. We are interested in the growth of the population of individuals with a specific choice function over time, and we write n_t for the number of such individuals in generation t. In general, we have

$$n_t = \sum_{i=1}^{n_{t-1}} \left(I_{it} x_{ait} + (1 - I_{it})\, x_{bit} \right),$$

where the sum runs over all individuals in generation $t - 1$. We assume that although all the individuals have the same function I, the random variable for each individual is independent of all the others. We also assume that the distributions Φ_{it} are independent and identically distributed

across individuals i and times t. Under these assumptions, the value of n_t can be expressed as

$$\begin{aligned} n_t &\overset{p}{=} n_{t-1}\left(\mathrm{E}\left[I_t x_{at}\right] + \mathrm{E}\left[\left(1-I_t\right)x_{bt}\right]\right) \\ &\overset{p}{=} n_{t-1}\left(\mathrm{E}\left[I_t\right]\mathrm{E}\left[x_{at}\right] + \mathrm{E}\left[1-I_t\right]\mathrm{E}\left[x_{bt}\right] + \mathrm{Cov}\left(I_t, x_{at}\right) + \mathrm{Cov}\left(1-I_t, x_{bt}\right)\right) \end{aligned} \tag{1}$$

where the expectations and covariances in (1) are calculated for a typical individual having offspring at time t. No subscript, i, is needed to index these individuals since all members of the population have the same expectation of outcomes and the same choice function I_t. The symbol $\overset{p}{=}$ denotes equivalence in probability, and this equivalence (1) follows from the Law of Large Numbers.[1] Introducing some new notation, we can rewrite (1) as

$$n_t \overset{p}{=} n_{t-1}\left(f\mu_{at} + (1-f)\mu_{bt} + \sqrt{f(1-f)}\left(\sigma_{at}\rho_{at} + \sigma_{bt}\rho_{bt}\right)\right), \tag{2}$$

where μ_{at} and μ_{bt} represent the common expected values for x_{ait} and x_{bit} for each individual i at time t, where σ_{at} and σ_{bt} represent the corresponding standard deviations, and where ρ_{at} and ρ_{bt} represent the common correlations at time t of each x_{ait} and x_{bit} with I_{it} and $1 - I_{it}$, respectively. Because all of these values are the same across all individuals having offspring at time t, the subscript i is not necessary in any of the terms in (2). It is also convenient to write

$$n_t \overset{p}{=} n_{t-1}\left(f\mu_{at} + (1-f)\mu_{bt} + \sigma_t\rho_t\sqrt{f(1-f)}\right), \tag{3}$$

where ρ_t is the common correlation of I_{it} with $y_{it} = x_{ait} - x_{bit}$ for each individual i having offspring at time t, and where σ_t is the common standard deviation of y_{it} for such individuals.

We use backward recursion to find that

$$n_T \overset{p}{=} n_0 \prod_{t=1}^{T}\left(f\mu_{at} + (1-f)\mu_{bt} + \sigma_t\rho_t\sqrt{f(1-f)}\right),$$

where n_0 is the number of individuals in the population at time $t = 0$. From this we deduce that

$$\frac{1}{T}\log n_T \overset{p}{\to} \mathrm{E}\left[\log\left(f\mu_{at} + (1-f)\mu_{bt} + \sigma_t\rho_t\sqrt{f(1-f)}\right)\right], \tag{4}$$

where the expectation is taken with respect to the continuous limit of the distribution of the random variable values over times t.[2]

[1]In particular, the sums over the sample population converge almost surely to the unrestricted means and covariances. This follows since the variance of each relevant random variable must be bounded, provided that there is an upper bound on the possible number of offspring a single individual may have.

[2]Note that to apply the Law of Large Numbers here we assume that the terms $\log\left(f\mu_{at} + (1-f)\mu_{bt} + \sigma_t\rho_t\sqrt{f(1-f)}\right)$ have bounded variance. This assumption is valid provided that the distribution of the argument of the logarithm does not have positive mass in arbitrarily small neighborhoods of zero.

It is convenient to introduce a new notation for the right-hand side of (4), namely

$$\alpha = \mathrm{E}\left[\log\left(f\mu_{at} + (1-f)\mu_{bt} + \sigma_t\rho_t\sqrt{f(1-f)}\right)\right]. \tag{5}$$

In what follows, we seek to identify the values of I that give rise to the maximum value for α, since individuals with such values will dominate the population over time in a sense made precise by the following proposition.

Proposition 1 *Suppose that two different choice functions, I_1 and I_2, give rise to values α_1 and α_2, with the property that $\alpha_1 > \alpha_2$. Individuals with the choice function I_1 will become exponentially more numerous over time, since*

$$\lim_{T\to\infty} \frac{n_T(I_2)}{n_T(I_1)} \xrightarrow{p} \lim_{T\to\infty} e^{T(\alpha_2-\alpha_1)} = 0.$$

In the following sections, we consider the parameters giving rise to maximal values for α under various specific assumptions about the nature of the distributions Φ_{it}.

2 The Case of No Intelligence

We say that a member of the population exhibits intelligence if its behavior correlates positively with outcomes, i.e., if $\rho > 0$. If $\rho = 0$, however, then we say that no intelligence is present.[3] In this situation, we can write $\alpha = \alpha(f)$, and we write f^* for the value of f that gives rise to the maximum value of α. Also, we can write the expression for α from (5) as

$$\alpha(f) = \mathrm{E}\left[\log\left(f\mu_{at} + (1-f)\mu_{bt}\right)\right].$$

The value f^* that maximizes this expression for α is characterized by the following proposition.

Proposition 2 *If intelligence is not present in a population, the growth-optimal behavior f^* is given by*

$$f^* = \begin{cases} 1 & \text{if } \mathrm{E}[\mu_{at}/\mu_{bt}] > 1 \text{ and } \mathrm{E}[\mu_{bt}/\mu_{at}] < 1 \\ \text{solution to (7)} & \text{if } \mathrm{E}[\mu_{at}/\mu_{bt}] \geq 1 \text{ and } \mathrm{E}[\mu_{bt}/\mu_{at}] \geq 1 \\ 0 & \text{if } \mathrm{E}[\mu_{at}/\mu_{bt}] < 1 \text{ and } \mathrm{E}[\mu_{bt}/\mu_{at}] > 1 \end{cases} \tag{6}$$

where f^* is defined implicitly in the second case of (6) by:

$$\mathrm{E}\left[\frac{\mu_{at}}{f^*\mu_{at} + (1-f^*)\mu_{bt}}\right] = \mathrm{E}\left[\frac{\mu_{bt}}{f^*\mu_{at} + (1-f^*)\mu_{bt}}\right] \tag{7}$$

[3]The case $\rho < 0$ would correspond to "intelligence" that leads to a less favorable outcome than no intelligence, and so we exclude this case from consideration.

and the expectations are taken with respect to the joint distributions across time t for μ_{at} and μ_{bt}, as these distributions are implied by the Φ_{it}.

Proof. The result can be seen by computing the first and second derivatives of α. Because the second derivative is strictly negative, there is exactly one maximum value obtained in the interval $f \in [0, 1]$. The values of the first derivative of $\alpha(f)$ at the endpoints of the interval are $\alpha'(0) = \mathrm{E}\left[\mu_{at}/\mu_{bt}\right] - 1$ and $\alpha'(1) = 1 - \mathrm{E}\left[\mu_{bt}/\mu_{at}\right]$. If $\alpha'(0)$ and $\alpha'(1)$ are both positive or both negative, then $\alpha(f)$ increases or decreases, respectively, throughout the interval and the maximum value is attained at $f = 1$ or $f = 0$, respectively. Otherwise, $f = f^*$ is the unique point in the interval for which $\alpha'(f) = 0$, where f^* is defined in (7), and it is at this point that $\alpha(f)$ attains its maximum value. The expression (6) summarizes the results of these observations for the various possible values of $\mathrm{E}\left[\mu_{at}/\mu_{bt}\right]$ and $\mathrm{E}\left[\mu_{bt}/\mu_{at}\right]$. Note that the case $\mathrm{E}\left[\mu_{at}/\mu_{bt}\right] \leq 1$ and $\mathrm{E}\left[\mu_{bt}/\mu_{at}\right] \leq 1$ is not considered because this set of inequalities implies that $\alpha'(0) \leq 0$ and $\alpha'(1) \geq 0$, which is impossible, since $\alpha''(f)$ is strictly negative. ∎

3 A Universal Measure and Cost of Intelligence

As we have noted, the case of no intelligence corresponds to no correlation, i.e., $\rho_t = 0$, while the case of intelligence corresponds to positive correlation, i.e., $\rho_t > 0$, with higher values representing more intelligence. The correlation ρ_t cannot necessarily assume any value in the range $[0, 1]$, however, and it is in fact constrained by the choice of f. More specifically, ρ_t can assume all values in the range $[0, \rho_{t,\max}(f)]$ but no values outside this range, where $\rho_{t,\max}(f)$ is a function dependent on f and the Φ_{it}. A precise value for $\rho_{t,\max}$ is calculated in Proposition 3, below. Because the upper bound for ρ_t depends upon f, the measure ρ_t is difficult to use as a universal representation of underlying intelligence. We therefore introduce the additional variable γ, defined as

$$\gamma = \frac{\rho_t}{\rho_{t,\max}(f)}. \tag{8}$$

This is a universal representation of intelligence in the sense that it represents the fraction of the maximum possible correlation achievable, and this fraction remains constant even as the maximum possible correlation varies with f.

In the case in which there is no variation in the Φ_{it} across time, then the values of ρ_t and $\rho_{t,\max}(f)$ are the same for all t, and we write these common value as ρ and $\rho_{\max}(f)$. This is the case we considered in our main paper, and there we simply used ρ as the measure of intelligence instead of γ, since the two measures are the same up to a constant rescaling factor that is common across all generations. For purposes of this Supplementary Information, however, we deal with the more general situation in which we must use γ instead of ρ as the universal measure of intelligence.

We suppose that a member of a population has a particular value of $f \in [0, 1]$ and a particular value of $\gamma \in [0, 1]$, and that these attributes are passed on to all offspring of an individual. In terms of f and γ, the expression for α in in (5) can be written

$$\alpha = \mathrm{E}\left[\log\left(f\mu_{at} + (1-f)\mu_{bt} + \sigma_t\gamma\rho_{t,\max}(f)\sqrt{f(1-f)}\right)\right]. \tag{9}$$

We also consider the possibility that γ has a cost, $c(\gamma)$, associated with it, and that once this cost is factored in, the expression for α becomes

$$\alpha = \mathrm{E}\left[\log\left(f\mu_{at} + (1-f)\mu_{bt} + \sigma_t\left(\gamma - c(\gamma)\right)\rho_{t,\max}(f)\sqrt{f(1-f)}\right)\right]. \tag{10}$$

We assume that $c(0) = 0$ and $c(\gamma) > 0$ for $\gamma > 0$. We also assume that $\gamma - c(\gamma) > 0$ for sufficiently small values of γ and that $\gamma - c(\gamma) < 0$ for values of γ sufficiently close to 1. Thus, at least some small amount of intelligence is beneficial, but high costs make the choice of $\gamma = 1$ prohibitively expensive. In addition, we make the further assumption that c is twice continuously differentiable and that $c'(\gamma) > 0$ and $c''(\gamma) > 0$. Because of this assumption, there is a unique value of γ^* that maximizes $\gamma - c(\gamma)$.

$$\gamma^* = \text{unique } \gamma \text{ such that } c'(\gamma) = 1. \tag{11}$$

It is convenient to introduce some additional notation related to the distribution of the y_{it}, which is given by the function Φ_{it}. We write φ_t for the probability that $y_{it} > 0$ for individuals in generation t, i.e.,

$$\varphi_t = \mathrm{Prob}(y_{it} > 0). \tag{12}$$

This value is thus the probability that choice a is superior to choice b in generation t. In addition, we write δ_t^+ and δ_t^- for the expected value of y_{it} conditional on either $y_{it} > 0$ or $y_{it} \leq 0$, respectively. That is,

$$\delta_t^+ = \mathrm{E}[y_{it}|y_{it} > 0] \quad \text{and} \quad \delta_t^- = \mathrm{E}[y_{it}|y_{it} \leq 0]. \tag{13}$$

The values of φ_t, δ_t^+, and δ_t^- are constant across all individuals in generation t because the functions Φ_{it} are independent and identical across individuals in generation t. In much of what follows, we also find it convenient to make the following assumption about the independence of y_{it} and I_{it}, conditional on the sign of y_{it}. This assumption may be violated in a fully general case, but it allows us to simplify our analysis and obtain more tractable formulas while still retaining a rich framework in which to operate.

A 1 *For all i and t, conditional on the sign of y_{it}, the distribution of y_{it} and the distribution of I_{it} are independent. Thus,*

$$\mathrm{E}[I_{it}y_{it}|y_{it} > 0] = \delta_t^+\,\mathrm{E}[I_{it}|y_{it} > 0] \quad \textit{and} \quad \mathrm{E}[I_{it}y_{it}|y_{it} \leq 0] = \delta_t^-\,\mathrm{E}[I_{it}|y_{it} \leq 0].$$

In other words, the value of I_{it} can only depend upon the sign of y_{it}, and thus the question of whether a or b is the superior choice, and not upon additional information about the degree of the superiority of one choice over the other.

Under assumption A1, and using Propositions 3 and 4, which are proven in Section 4 below, we can rewrite (10) as

$$\alpha = \mathrm{E}\left[\log\left(f\mu_{at} + (1-f)\mu_{bt} + \left(\delta_t^+ - \delta_t^-\right)\left(\gamma - c(\gamma)\right)\left(\min(f,\varphi_t) - f\varphi_t\right)\right)\right]. \tag{14}$$

We seek the values of f and γ that maximize α, as defined in (14). If the optimal value occurs when $f = 0$ or $f = 1$, then the amount of intelligence is clearly irrelevant, since the term involving

intelligence vanishes and behavior is simply deterministic. If the optimal value occurs when $f \in (0,1)$, then the optimal amount of intelligence is clearly $\gamma = \gamma^*$, as defined above. The nature of the optimal choice of f is derived in Propositions 5 and 6, in the case of no systematic variation across generations, and Proposition 7, in the general case.

4 Upper Bound on Correlation

In this section we consider the restrictions on the possible values for ρ_t. The value of ρ_t is subject to constraints that depend upon the nature of the distributions $\Phi_{it}(x_a, x_b)$, as well as on the value of f for the population. The next proposition makes this dependence clear, when assumption A1 holds.

Proposition 3 *Under assumption A1, the value of ρ_t is given by*

$$\rho_t = \mathrm{Corr}\left(I_{it}, y_{it}\right) = \mathrm{Corr}\left(I_{it}, H\left(y_{it}\right)\right) \left(\frac{\delta_t^+ - \delta_t^-}{\sigma_t / \sqrt{\varphi_t(1-\varphi_t)}}\right), \tag{15}$$

where $H(y_{it})$ is the Heaviside function, which is 1 when $y_{it} > 0$ and 0 otherwise. The values of δ_t^+, δ_t^-, and φ_t are as defined in (13) and (12). The correlation between I_{it} and $H\left(y_{it}\right)$ may be also be written as

$$r_t \stackrel{def}{=} \mathrm{Corr}\left(I_{it}, H\left(y_{it}\right)\right) = \frac{\left(\pi_t - \varphi_t f\right)}{\sqrt{\varphi_t(1-\varphi_t)}\sqrt{f(1-f)}}, \tag{16}$$

where

$$\pi_t = \mathrm{Prob}\left(I_{it} = 1 \text{ and } y_{it} > 0\right). \tag{17}$$

The value of r_t and that of π_t are the same for each individual i in a given generation t.

Proof. The result follows directly from the definition of correlation. We have

$$\begin{aligned}
\mathrm{Corr}\left(I_{it}, y_{it}\right) &= \frac{\mathrm{E}\left[I_{it} y_{it}\right] - \mathrm{E}\left[I_{it}\right]\mathrm{E}\left[y_{it}\right]}{\mathrm{Std}\left(y_{it}\right)\mathrm{Std}\left(I_{it}\right)} \\
&= \frac{\pi_t \delta_t^+ + (f - \pi_t)\delta_t^- - f\left(\varphi_t \delta_t^+ + (1-\varphi_t)\delta_t^-\right)}{\sigma_t \sqrt{f(1-f)}} \\
&= \left(\pi_t - \varphi_t f\right) \frac{\delta_t^+ - \delta_t^-}{\sigma_t \sqrt{f(1-f)}} \\
&= \left(\frac{\pi_t - \varphi_t f}{\sqrt{f(1-f)}\sqrt{\varphi_t(1-\varphi_t)}}\right)\left(\frac{\delta_t^+ - \delta_t^-}{\sigma_t / \sqrt{\varphi_t(1-\varphi_t)}}\right) \\
&= \mathrm{Corr}\left(I_{it}, H\left(y_{it}\right)\right)\left(\frac{\delta_t^+ - \delta_t^-}{\sigma_t / \sqrt{\varphi_t(1-\varphi_t)}}\right),
\end{aligned}$$

which proves (15).

The value of π_t does not depend upon the choice of individual i because the functions Φ_{it} and I_{it} are independent and identically distributed across individuals in generation t. The lack of dependence of r_t on the choice of individual i within a given generation can be seen by noting that

$$r_t = \mathrm{Corr}\left(I_{it}, H\left(y_{it}\right)\right) = \frac{\pi_t - \varphi_t f}{\sqrt{f(1-f)}\sqrt{\varphi_t(1-\varphi_t)}},$$

and observing that the right-hand side of this equation does not depend upon the choice of individual i. ∎

An implication of the formula in (15) is that the possible values for ρ_t cannot necessarily be arbitrarily close to 1. The range of possible values for ρ_t is made more precise by the following proposition.

Proposition 4 *Under assumption A1, the range of possible values for π_t when intelligence is present is*

$$f\varphi_t < \pi_t \leq \min\left(f, \varphi_t\right). \tag{18}$$

The corresponding range of possible values for r_t is

$$0 < r_t \leq r_{t,\max}, \quad \textit{where} \quad r_{t,\max} = \frac{\max\left(f, \varphi_t\right) - \varphi_t f}{\sqrt{f(1-f)}\sqrt{\varphi_t(1-\varphi_t)}}. \tag{19}$$

The inequality $r_t < 1$ holds whenever $f \neq \varphi_t$. The corresponding range of possible values for ρ_t is

$$0 < \rho_t \leq \rho_{t,\max}, \quad \textit{where} \quad \rho_{t,\max} = r_{t,\max}\frac{\delta_t^+ - \delta_t^-}{\sigma_t/\sqrt{\varphi_t(1-\varphi_t)}}. \tag{20}$$

The inequality $\rho_{t,\max} < 1$ holds unless $r_t = 1$ and both $\mathrm{E}\left[y_{it}^2 | y_{it} > 0\right] = \left(\mathrm{E}\left[y_{it}^2 | y_{it} > 0\right]\right)^2$ and $\mathrm{E}\left[y_{it}^2 | y_{it} \leq 0\right] = \left(\mathrm{E}\left[y_{it}^2 | y_{it} \leq 0\right]\right)^2$.

Proof. From the definition of π_t in (17), it is clear that π must be bounded above by $\min\left(f, \varphi_t\right)$. Also, because intelligence only occurs when $\rho_t > 0$, the formulas in (15) and (16) show that it must also be the case that π_t is bounded below by $f\varphi_t$, and this suffices to prove (18).

The range of possible values for r_t can be derived by substituting the limits of the possible range for π_t into the expression for r_t in (16), and the range of possible values for ρ_t follows from (15).

To obtain an upper bound on $\rho_{t,\max}$, it is useful to proceed by first deriving a lower bound for σ_t. Note that Hölder's Inequality shows that

$$\sigma_t^2 = \mathrm{E}\left[y_{it}^2\right] - (\mathrm{E}\left[y_{it}\right])^2 = \varphi_t \,\mathrm{E}\left[y_{it}^2|y_{it} > 0\right] + (1-\varphi_t)\,\mathrm{E}\left[y_{it}^2|y_{it} \le 0\right]$$

$$- \left(\varphi_t \,\mathrm{E}\left[y_{it}|y_{it} > 0\right] + (1-\varphi_t)\,\mathrm{E}\left[y_{it}|y_{it} \le 0\right]\right)^2$$

$$\ge \varphi_t \left(\mathrm{E}\left[y_{it}|y_{it} > 0\right]\right)^2 + (1-\varphi_t)\left(\mathrm{E}\left[y_{it}^2|y_{it} \le 0\right]\right)^2$$

$$- \left(\varphi_t \,\mathrm{E}\left[y_{it}|y_{it} > 0\right] + (1-\varphi_t)\,\mathrm{E}\left[y_{it}|y_{it} \le 0\right]\right)^2$$

$$= \varphi_t(1-\varphi_t)\left(\delta_t^+ - \delta_t^-\right)^2.$$

The expectations are all taken with respect to a particular individual within generation t and are also independent of the specific choice of individual within the generation. Also, the conditions for equality in Hölder's inequality show that equality only holds for our lower bound on σ_t^2 when both $\mathrm{E}\left[y_{it}^2|y_{it} > 0\right] = \left(\mathrm{E}\left[y_{it}^2|y_{it} > 0\right]\right)^2$ and $\mathrm{E}\left[y_{it}^2|y_{it} \le 0\right] = \left(\mathrm{E}\left[y_{it}^2|y_{it} \le 0\right]\right)^2$. The lower bound on σ_t^2 can be re-written as

$$\frac{\delta_t^+ - \delta_t^-}{\sigma_t/\sqrt{\varphi_t(1-\varphi_t)}} \le 1,$$

and this the upper bound on $\rho_{t,\max}$ described in the proposition. ∎

5 Intelligence and No Variation Across Generations

We now consider the case in which there may be intelligence, so that it is possible to have $\gamma > 0$, but we assume that there is no variation in the distribution of possible outcomes across generations. Thus, we have $\mu_{at} = \mu_a$, $\mu_{bt} = \mu_b$, $\delta_t^+ = \delta^+$, $\delta_t^- = \delta^-$, and $\varphi_t = \varphi$. In this case, we can write the expression for α from (9) as

$$\alpha = \log\left(f\mu_a + (1-f)\mu_b + (\gamma - c(\gamma))\left(\min(\varphi, f) - \varphi f\right)\left(\delta^+ - \delta^-\right)\right). \quad (21)$$

Note that we do not take the expectation of the logarithm in this expression for α, since the value of the logarithm is constant across generations under the current assumptions. The values of f and γ that maximize α in the case in which intelligence is costless (so that $c(\gamma) \equiv 0$) are characterized by the following proposition.

Proposition 5 *Under assumption A1, and under the further assumptions that intelligence has no cost and that there is no variation in outcome possibilities across generations, the values of f and γ at which α is maximized are $f = \varphi$ and $\gamma = 1$, provided that $\varphi \in (0, 1)$. If φ is either 0 or 1, then α is maximized when $f = \varphi$, and the value of γ is irrelevant.*

Proof. The expression for α in (21) is maximized when the argument of the logarithm is maximized, and this can be written

$$e^\alpha = f\mu_a + (1-f)\mu_b + \gamma\left(\min(\varphi, f) - \varphi f\right)\left(\delta^+ - \delta^-\right).$$

The partial derivative of e^α with respect to γ is

$$\frac{\partial e^\alpha}{\partial \gamma} = (\min(\varphi, f) - \varphi f)\left(\delta^+ - \delta^-\right),$$

and this value is greater than 0 provided that $f \notin \{0,1\}$, since $\delta^+ - \delta^- > 0$. Thus, if $f \notin \{0,1\}$, e^α is a strictly increasing function of γ, and the maximum value of e^α for any fixed value of $f \in (0,1)$ is obtained when $\gamma = 1$.

The partial derivative of e^α with respect to f is

$$\frac{\partial e^\alpha}{\partial f} = \varphi(1-\gamma)\delta^+ + (1 - \varphi(1-\gamma))\delta^-,$$

when $f > \varphi$, and

$$\frac{\partial e^\alpha}{\partial f} = (1 - (1-\varphi)(1-\gamma))\delta^+ + (1-\varphi)(1-\gamma)\delta^-,$$

when $f < \varphi$. When $\gamma = 1$, the expressions for $\partial e^\alpha / \partial f$ becomes simply δ^- or δ^+, when $f > \varphi$ and $f < \varphi$, respectively. Because $\delta^- < 0$ and $\delta^+ > 0$, e^α obtains its maximum for $f \in (0,1)$ is at $f = \varphi$ and $\gamma = 1$.

If φ is 0 or 1, then the value of f that maximizes α and e^α is clearly $f = \varphi$, and the value of γ is irrelevant, since intelligence is irrelevant for an optimal outcome in this situation. ∎

Proposition 5 shows that, when $c(\gamma) \equiv 0$, more intelligence, i.e., a higher γ value, is always desirable, except in situations in which behavior is completely deterministic, i.e., f is equal to 0 or 1. This result makes sense when intelligence is costless, but to make the situation more realistic, we also consider the situation in which an intelligence level of γ is associated with a cost $c(\gamma) \not\equiv 0$ of the type described in Section 3. The next proposition characterizes the values of f and γ that maximize α when there is no variation across generations and when there is such a cost to intelligence.

Proposition 6 *Under assumption A1, and under the further assumptions that there is no variation in outcome possibilities across generations and that there is a cost $c(\gamma)$ of intelligence of the type described in Section 3, the values of f and γ that maximize α are characterized in the following way. If $\mu_b > \mu_a$ and $\varphi \in (0,1)$, then*

$$f^* = \begin{cases} \varphi & \textit{if } \gamma^* - c(\gamma^*) > \frac{\mu_b - \mu_a}{\delta^+ + \mu_b - \mu_a}; \\ [0, \varphi] & \textit{if } \gamma^* - c(\gamma^*) = \frac{\mu_b - \mu_a}{\delta^+ + \mu_b - \mu_a}; \textit{ and} \\ 0 & \textit{if } \gamma^* - c(\gamma^*) < \frac{\mu_b - \mu_a}{\delta^+ + \mu_b - \mu_a}. \end{cases} \tag{22}$$

Here γ^* is as defined in (11). If $\mu_b < \mu_a$ and $\varphi \in (0,1)$, then

$$f^* = \begin{cases} \varphi & \text{if } \gamma^* - c(\gamma^*) > \frac{\mu_a - \mu_b}{\mu_a - \mu_b - \delta^-}; \\ [\varphi, 1] & \text{if } \gamma^* - c(\gamma^*) = \frac{\mu_a - \mu_b}{\mu_a - \mu_b - \delta^-}; \text{ and} \\ 1 & \text{if } \gamma^* - c(\gamma^*) < \frac{\mu_a - \mu_b}{\mu_a - \mu_b - \delta^-}. \end{cases} \tag{23}$$

If $\mu_a = \mu_b$ then $f^* = \varphi$, and if $\varphi \in \{0,1\}$, then $f^* = \varphi$. In all cases for which $f^* \notin \{0,1\}$, the optimal choice of γ is γ^*. If, however, $f^* \in \{0,1\}$, then intelligence is unimportant, and the choice of γ does not matter.

Proof. The proof follows from a straightforward analysis of the partial derivatives of e^α with respect to γ and f. The derivative of e^α with respect to γ is

$$\frac{\partial e^\alpha}{\partial \gamma} = (1 - c'(\gamma)) \left(\min(\varphi, f) - \varphi f\right) \left(\delta^+ - \delta^-\right),$$

and for any value of $f \in (0,1)$, this partial derivative is zero exactly when $\gamma = \gamma^*$. Thus, if the optimal value of f is in the interior of the interval $[0,1]$, the optimal value of γ is γ^*.

When $\gamma = \gamma^*$, the partial derivative of e^α with respect to f can be written

$$\frac{\partial e^\alpha}{\partial f} = \mu_a - \mu_b + (\delta^+ - \delta^-)(\gamma^* - c(\gamma^*))(1 - \varphi), \tag{24}$$

when $f < \varphi$, and

$$\frac{\partial e^\alpha}{\partial f} = \mu_a - \mu_b + (\delta^+ - \delta^-)(\gamma^* - c(\gamma^*))(-\varphi), \tag{25}$$

when $f > \varphi$. When $\mu_b > \mu_a$ and $\varphi \in (0,1)$, the expression in (25) is always negative, and so e^α is decreasing in f in the region $f \in [\varphi, 1]$. Also, the sign of (24) is positive, zero, or negative, according to whether $\gamma^* - c(\gamma^*)$ is larger, equal to, or less than, respectively, the value of $\frac{\mu_b - \mu_a}{\delta^+ + \mu_b - \mu_a}$. In these three situations, the function e^α is increasing, constant, or decreasing, respectively, in the region $f \in [0, \varphi]$. These observations lead directly to the results in (22).

The remaining results of the proposition follow from similar analysis of the partial derivative of e^α with respect to f in the various cases described. ∎

6 Example of Optimal Choice with Intelligence

In this section, we provide an illustrative example of our model in the case of no variation across generations and the possibility of intelligence in behavior.

For purposes of our example, we assume a cost function of a particular type, namely

$$c(\gamma) = \kappa \frac{\gamma^2}{1 - \gamma},$$

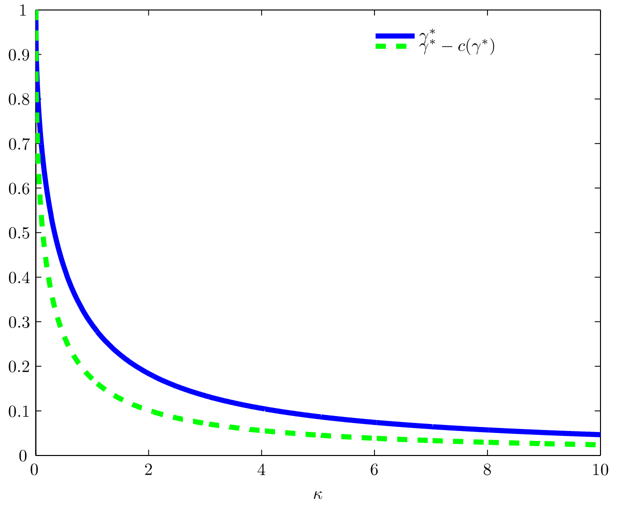

Figure 1: Values of γ^* and $\gamma^* - c(\gamma^*)$ as functions of κ, the cost of intelligence parameter in (26).

where $\kappa > 0$ is a parameter that can be chosen higher to indicate a greater cost to intelligence, or lower to indicate the reverse situation. This function $c(\gamma)$ can also be written

$$c(\gamma) = \kappa\left(-\gamma - 1 + \frac{1}{1-\gamma}\right), \tag{26}$$

and it is straightforward to check that it satisfies all of our requirements for a cost function for $\gamma \in [0,1]$. Specifically, $\gamma - c(\gamma) > 0$ for small values of γ, and $\gamma - c(\gamma) < 0$ for values of γ sufficiently close to 1. Also, $c(\gamma)$ is twice continuously differentiable, is increasing, and is convex. For this cost function, the value of γ^* defined in (11) can be written

$$\gamma^* = 1 - \sqrt{\frac{\kappa}{1+\kappa}}.$$

Also, the value of $\gamma^* - c(\gamma^*)$, which is needed to determine the cases specified in Proposition 6, can be written

$$\gamma^* - c(\gamma^*) = 1 + 2\left(\kappa - \sqrt{\kappa(1+\kappa)}\right).$$

The values of γ^* and $\gamma^* - c(\gamma^*)$ are plotted as functions of κ in Figure 1.

The result of Proposition 6 is illustrated in Figure 2. We assume that $\mu_b > \mu_a$, and we use the horizontal axis to indicate the size of the ratio $r = \delta^+/(\mu_b - \mu_a)$. We use the vertical axis to indicate the value of κ. For any r and κ values, Proposition 6 can be used to determine the optimal f value, namely f^*. The value of f^* is either 0 or φ, except when $\gamma^* - c(\gamma^*) = 1/(1+r)$, and in this special case the value of f^* may be anywhere between 0 and φ. The deterministic value 0 is possible while the deterministic value 1 is not simply because we have assumed that $\mu_b > \mu_a$. As the figure indicates, a sufficiently high cost of intelligence, as indicated by a high κ value, corresponds to the deterministic choice $f^* = 0$ and no use of intelligence. When intelligence has a low enough cost for a given ratio value, however, the optimal choice is $f^* = \varphi$, which is the same frequency for f as occurs in probability matching.

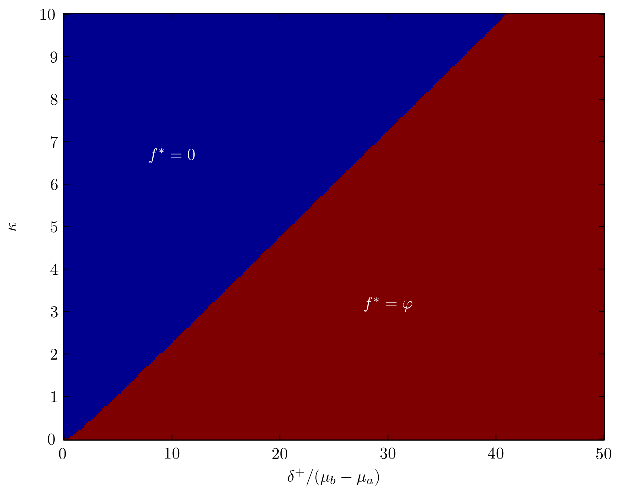

Figure 2: Values of f^* for particular values of κ and $r = \delta^+/(\mu_b - \mu_a)$. The region toward the upper left corresponds to relatively costly intelligence and deterministic behavior of the form $f^* = 0$. The region toward the lower right corresponds to relatively cheap intelligence and probability matching of the form $f^* = \varphi$. On the line between the two large regions, any value for f^* between 0 and φ is optimal.

7 Intelligence and Variation Across Generations

The final case we consider is the one in which individuals may be intelligent and in which there may be variation in outcomes over time. The following proposition describes nature of the optimal choice of f and γ in this setting under certain assumptions.

Proposition 7 *Under assumption A1, and under the further assumptions that the distribution of φ_t is smooth and that intelligence has a cost of the type described in Section 3, the maximal value of α occurs when $\gamma = \gamma^*$. In addition, if the optimal choice of f is in the interior of the interval $[0, 1]$, then this choice is defined implicitly by the equation*

$$\mathrm{E}\left[\frac{\mu_{at} - \mu_{bt} + (\gamma^* - c(\gamma^*))\left(\delta_t^+ - \delta_t^-\right)(H(\varphi_t - f) - \varphi_t)}{f^*\mu_{at} + (1 - f^*)\mu_{bt} + (\gamma^* - c(\gamma^*))\left(\delta_t^+ - \delta_t^-\right)(\min(\varphi_t, f) - \varphi_t f)}\right] = 0,$$

where H is the Heaviside function, and where the expectations are taken with respect to the joint distributions across time t for μ_{at} and μ_{bt}, as these distributions are implied by the Φ_{it}.

Proof. The optimality of the choice $\gamma = \gamma^*$ follows from consideration of the derivative of α with respect to γ. The optimality of f described in the proposition follows from the fact that the second derivative of α with respect to f is continuous and strictly negative, since φ_t has a smooth distribution. Also, because the optimal value of f is assumed to occur in the interior of the interval $[0, 1]$, it follows that this value is the unique place at which the derivative with respect to f vanishes, when $\gamma = \gamma^*$. ∎

Acknowledgements

We thank Henry Cohn, Doyne Farmer, Simon Levin, Tommy Poggio, Arthur Robson, and Jon Wilkins for helpful comments and discussion. The views and opinions expressed in this article are those of the authors only, and do not necessarily represent the views and opinions of: the individuals acknowledged above, Northwestern University, MIT, AlphaSimplex Group, LLC, or any of their affiliates and employees. Research support from the MIT Laboratory for Financial Engineering and the Northwestern University School of Law Faculty Research Program is gratefully acknowledged.
